# FoxO3a (Forkhead Box O3a) Deficiency Protects Idiopathic Pulmonary Fibrosis (IPF) Fibroblasts from Type I Polymerized Collagen Matrix-Induced Apoptosis via Caveolin-1 (cav-1) and Fas

**DOI:** 10.1371/journal.pone.0061017

**Published:** 2013-04-08

**Authors:** Richard Seonghun Nho, Mark Peterson, Polla Hergert, Craig A. Henke

**Affiliations:** Department of Medicine, University of Minnesota, Minneapolis, Minnesota, United States of America; AMS Biotechnology, United Kingdom

## Abstract

Idiopathic Pulmonary Fibrosis is a lethal fibrotic disease characterized by the unrelenting proliferation and persistence of fibroblasts in a type I collagen-rich matrix that result in an expanding reticular network of fibrotic tissue. However, the underlying mechanism responsible for the persistence of myofibroblasts in IPF remains unclear. During normal tissue repair, unwanted fibroblasts are eliminated during collagen-matrix contraction by a mechanism whereby high PTEN activity suppresses Akt. We have previously found that FoxO3a, a transcriptional activator of apoptosis-inducing proteins, is inactivated in IPF fibroblasts resulting from aberrantly high PI3K/Akt activity due to inappropriately low PTEN activity. Here we demonstrate that this low FoxO3a activity confers IPF fibroblasts with resistance to collagen-mediated apoptosis. We show that the mechanism by which low FoxO3a activity confers IPF fibroblasts with an apoptotic resistant phenotype involves suppression of Fas expression as a result of down regulation of cav-1 expression via a PTEN/Akt-dependent pathway. We demonstrate that PTEN over-expression or Akt inhibition increases FoxO3a expression in IPF fibroblasts, resulting in up-regulation of caveolin-1. We show that FoxO3a binds to the cav-1 promoter region and ectopic expression of FoxO3a transcriptionally increases cav-1 mRNA and protein expression. In turn, we show that overexpression of caveolin-1 increases Fas levels and caspase-3/7 activity and promotes IPF fibroblast apoptosis on polymerized type I collagen. We have found that the expression of caveolin-1, Fas and cleaved caspase-3 proteins in fibroblasts within the fibroblastic foci of IPF patient specimens is low. Our data indicate that the pathologically altered PTEN/Akt axis inactivates FoxO3a down-regulating cav-1 and Fas expression. This confers IPF fibroblasts with an apoptosis-resistant phenotype and may be responsible for IPF progression.

## Introduction

Idiopathic pulmonary fibrosis (IPF) is a chronic and progressive lung disorder of unknown etiology [1]–[3]. Currently there is no proven treatment for IPF, and the pathogenesis of this deadly disease is not well understood [4], [5]. IPF is characterized by unrelenting proliferation of fibroblasts with deposition of type I collagen within the alveolar wall resulting in scarred non-functional airspaces, hypoxia, and death by asphyxiation [6]–[8]. When normal fibroblasts interact with polymerized type I collagen via α2β1 integrin, PTEN activity is maintained in a range that suppresses the PI3K/Akt proliferation signal pathway [9]. This provides an effective physiologic mechanism to restrain fibroblast proliferation after tissue injury. In contrast, we have found that when IPF fibroblasts interact with polymerized collagen, α2β1 integrin levels are abnormally low resulting in pathologic activation of the PI3K/Akt due to inappropriately low PTEN function [9]–[12]. This enables IPF fibroblasts to escape the powerful negative regulation of proliferation normally exerted by a type I collagen rich environment [11]–[12].

The FoxO3a transcription factor controls the expression of proteins regulating both the cell cycle and cell viability. Active FoxO3a functions as a powerful inhibitor of the cell cycle and also promotes apoptosis [13], [21]. Importantly, recent work has linked aberrant suppression of FoxO3a activity with several human diseases including cancer progression [14]–[17]. We have discovered that inappropriately low FoxO3a activity plays a critical role in conferring IPF fibroblasts with their pathological phenotype [11]. Studies have demonstrated that FoxO3a activity is inhibited when Akt phosphorylates the ser 253 residue of FoxO3a, thus promoting transport of FoxO3a from the nucleus to the cytoplasm [18]–[20]. In this regard, we have found that FoxO3a activity is pathologically low when IPF fibroblasts interact with a type I collagen-rich matrix due to high Akt activity. This low FoxO3a function facilitates IPF fibroblast proliferation on polymerized collagen.

During normal tissue repair, excess fibroblasts are eliminated by apoptosis. The mechanism involves collagen contraction-mediated activation of PTEN thereby suppressing phosphorylated Akt levels [9], [10]. However, IPF is characterized by the persistence of fibroblasts in the type I collagen-rich fibrotic matrix, suggesting that IPF fibroblasts may display a resistant phenotype to collagen-mediated apoptosis. In this respect, prior work has found that IPF fibroblasts are resistant to Fas-ligand induced apoptosis due to low Fas expression, but the mechanism for low Fas expression in IPF is unclear. Importantly, prior work indicates that FoxO3a promotes cell apoptosis in part by up-regulating Fas expression [21]. Together, these observations suggested to us that pathologically low FoxO3a function in IPF fibroblasts may decrease Fas expression thereby maintaining their viability on collagen matrix via resistance to Fas-mediated apoptosis. Furthermore, recent studies have demonstrated that caveolin-1 (cav-1) regulates the Fas-mediated apoptotic pathway [36], by regulating Fas expression levels. Cav-1 is a main constituent of cellular membrane structures termed caveolae [25] and low cav-1 expression results in reduced Fas membrane expression. We have found that cav-1 expression is abnormally low in IPF fibroblasts interacting with polymerized collagen [12]. Since both low levels of cav-1 and FoxO3a can reduce Fas expression, this suggested a possible link between cav-1 and FoxO3a, and Fas expression. Here, we demonstrate that when IPF fibroblasts interact with type I collagen, low FoxO3a transcriptionally suppresses cav-1. Sequence analysis revealed that the cav-1 promoter contains several putative FoxO3a consensus binding sites and we found that FoxO3a binds to the cav-1 promoter region. We further investigated the FoxO3a/cav-1 axis in regulating IPF fibroblast viability on collagen. We demonstrate that over-expression of FoxO3a or cav-1 promotes IPF fibroblast apoptosis on collagen by increasing Fas protein expression. Furthermore, we demonstrate that caspase activity and apoptosis is attenuated in IPF fibroblasts over-expressing FoxO3a or cav-1 in the presence of Fas siRNA. Importantly, immunohistochemical analysis of IPF lung tissue demonstrated that cleaved caspase-3, cav-1 and Fas expression were low or absent while inactive FoxO3a levels were high in most fibroblasts within IPF fibroblastic foci. Taken together, our work strongly suggests that during IPF fibroblast interaction with polymerized collagen, inappropriately low PTEN function aberrantly activates Akt, which suppresses FoxO3a activity. Low FoxO3a activity transcriptionally suppresses cav-1 expression and results in low Fas expression thereby conferring IPF fibroblasts with an apoptotic-resistant phenotype. Our study reveals that a pathological PTEN/Akt/FoxO3a/cav-1/Fas pathway protects IPF fibroblasts from polymerized collagen-induced apoptosis thereby facilitating their persistence in the collagen-rich matrix and suggests that a therapeutic approach targeting this altered pathway may limit IPF progression.

## Materials and Methods

### Ethics Statement

This study involves the analysis of human IPF patient specimens. Primary fibroblast lines were obtained from unused, existing pathological human tissue samples, and therefore is exempt (exemption 4). Tissue samples were stripped of all identifiers and designated as waste. All patients underwent procedures for diagnostic or therapeutic procedures. Written informed consent was obtained on all patients prior to the procedure being performed. Use of human tissues was approved by the Institutional Review Board (IRB) at the University of Minnesota.

### Human Subjects

Cell lines were derived from lungs removed at the time of transplantation or death. The diagnosis of IPF was supported by history, physical examination, pulmonary function tests, and typical high resolution chest computed tomography findings of IPF. In all cases, the diagnosis of IPF was confirmed by microscopic analysis of lung tissue and demonstrated the characteristic morphological findings of usual interstitial pneumonia. All patients fulfilled the criteria for the diagnosis of IPF as established by the American Thoracic Society (ATS) and the European Respiratory Society (ERS). For this study, new primary IPF fibroblast lines were generated as tissue became available. To reduce technical variability, we routinely utilize cells between passages 5 and 7 because of concern that the phenotype of the cells is altered at higher passage. To address concerns of biological variability, we studied 8 IPF cell lines and 6 control cells lines.

### Cell Culture and Type I Collagen Matrices

8 IPF primary human lung fibroblast lines were established and analyzed for this study. Six control primary adult human lung fibroblast lines were established from histologically normal lung tissue distant from carcinoid tumor or radiation-induced fibrotic lung tissue or anatomically normal lung tissue not used at the time of transplantation. Primary lung fibroblast lines were generated by explant culture and cultured in high glucose DMEM containing 10% FCS. Fibroblasts were used between passages five and seven. Cells were characterized as fibroblasts as previously described [10]. FoxO3a+/+ and FoxO3a−/− mouse embryonic fibroblasts were kindly obtained from Dr Noboru Motoyama, National Center for Geriatrics and Gerontology, Japan. These cells were maintained in DMEM +10% FBS, 1% penicillin, and 1% streptomycin. Type I collagen solution was obtained from Advanced BioMatrix, CA. Three-dimensional polymerized collagen matrices (final concentration = 2 mg/ml) were prepared by neutralizing the collagen solution with a one-sixth volume of 6×DMEM medium and diluting to a final volume with 1×DMEM, and incubating the solution at 37°C for 3 to 4 h before use.

### Cell Viability and Caspase Assay

To precisely measure viable cells on collagen matrix, Cell-Titer-Blue Cell Viability Assay (Promega, WI) was used. Briefly, 3×10^4^ of control or IPF fibroblasts were cultured on type I polymerized collagen coated 96 well plates in 100 µl of serum free medium as a function of time. 20 µl of Cell Titer Blue reagent was then added to each well and further incubated for 1 to 2 h. Cell viability was measured by a 96 well plate reader with a fluorescence filter set 560(Ex)/590(Em). For caspase-3/7 activity assay, Apo-ONE Homogeneous Caspase-3/7 assay kit was used (Promega, WI). 3×10^4^ of control or IPF fibroblasts in 100 µl of serum free DMEM buffer cultured on polymerized collagen on 96 well plates were incubated with 100 µl of the mixture of caspase substrate and Apo-one caspase-3/7 buffer for 12 h. The fluorescence of each well with a filter set 485(Ex)/527(Em) was used to measure caspase-3/7 activity in samples.

### Antibodies, siRNA, Sequence Analysis, ChIP and Luciferase Assay

For Western analysis, PTEN, p-Akt (s473), cav-1, Fas antibodies were obtained from Cell Signaling Technologies, MA. GAPDH and actin antibodies and FoxO3a antibody for immunoprecipitation for ChIP assay were purchased from Santa Cruz biotechnologies. FoxO3a and inactive FoxO3a (S253 phosphorylated FoxO3a) antibodies for Western analysis were obtained from Millipore, MA and Cell Signaling Technologies, MA. Anti-human Fas activating clone CH11 was also obtained from Millipore. Immunohistochemical analysis of myofibroblasts within the fibroblastic foci of IPF patient specimens was performed using an α smooth muscle actin antibody (Vector Laboratories, CA). Cav-1, Fas siRNA and non-coding scrambled control siRNA were purchased from Life Technologies and Santa Cruz Biotechnologies, respectively using X-treme GENE siRNA transfection reagent (Roche Applied Science) according to manufacturer’s protocol. At 24 h post transfection, fibroblasts were cultured on polymerized collagen in serum free medium and assay was performed as described. For quantitative RT-PCR assay, total RNA was extracted from cells using Trizol (Life Technologies, NY) according to manufacturer’s protocol. cDNA was prepared using the Taqman Reverse Trascription kit (Life Technologies) with random hexamer primers and 1 µg of total RNA. Human forward 18S rRNA (5′-CAG CCA CCC GAG ATTT GAG CA 3′), human reverse 18S rRNA (5′-TAG TAG CGA CGG GCG GTG TG 3′), mouse forward 18S rRNA (5′-AGG GGA GAG CGG GTA AGA GA-3′), mouse reverse 18S rRNA (GGA CAG GAC TAG GCG GAA CA-3′), human forward cav-1 (5′- TGA CTG AGA AGC AAG TGT ATG ACG-3′), human reverse cav-1 (5′- GCA GAA GGT ATG GAC GTA GAT-3′), mouse forward cav-1 (5′-TGA CTG AGA AGC AAG TGT ATG ACG-3′), mouse reverse cav-1 (5′-GCA GAA GGT ATG GAC GTA GAT-3′) were prepared from the Biomedical Genomic Center at the University of Minnesota. Quantitative PCR was performed using a Light Cycler FastStart DNA Master^plus^ SYBR Green I Kit. Samples were quantified at the log-linear portion of the curve using LightCycler analysis software and compared to an external calibration standard carve. Cav-1 levels were normalized to 18S rRNA. Quantitative PCR parameters were as follows. Mouse 18SrRNA, 60°C annealing, 10 second extension for 40 cycles; mouse cav-1, 56°C annealing, 15 second extension for 40 cycles; human 18SrRNA, 62°C annealing, 11 second extension for 40 cycles; human cav-1, 56°C annealing, 15 second extension for 40 cycles. For the analysis of putative FoxO3a binding site on the cav-1 promoter region, MATCH program (version 1.0) was used. This software is designed to search potential binding sites for transcription factor nucleotide sequences using the library of mononucleotide weight matrices from TRANSFACT 6.0. The search is performed using 0.75 and 0.70 as cutoffs for core and matrix similarity, respectively using MATCH program. For the confirmation of FoxO3a binding to the cav-1 promoter, 2×10^5^ IPF fibroblasts were infected with 1×10^6^ PFU of adenovirus expressing wild type FoxO3a and agarose ChiP kit from Pierce was used according to manufacturer’s protocol. After crosslinking and micrococcal nuclease digestion steps, FoxO3a bound complex was immunoprecipitated with or without FoxO3a antibody (2 µg). Rabbit IgG was also used as a negative control. After DNA was recovered, C1 forward primer containing putative FoxO3a binding sites (i.e. GAAAACA, GAAAATT, GTAAAATA), 5′-CTC CAC CCC TGC TGA GAT GAT-3′ and C1 reverse primer 5′-AGT GAG AAC GTT GTC CCG CGC TGG-3′ were used for ChIP-PCR. PCR was carried out with 2 µl of recovered DNA using 0.25 µM of C1 primers as follows. 94°C for 7 min for 1 cycle, then 94°C for 30s, 55°C for 40 s and 72°C for 40 s for 35 cycles and 72°C for 1 cycle for 7 min. Amplified products were analyzed using 2% agarose gel. For the analysis of FoxO3a dependent transcriptional regulation, a luciferase reporter construct that contains Forkhead consensus binding sites (FHRE-Luc) were obtained from Addgene, MA. 2×10^5^ IPF and control fibroblasts expressing wild type FoxO3a, dominant negative FoxO3a or empty vector were transfected with 2 µg of pGL3-FHRE-Luc plasmid using FuGene HD transfection reagent (Roche, IN). Cells were then cultured on polymerized collagen in serum free medium for 24 h and lysed in 250 µl of lysis buffer. The luciferase activity of one fifth of the samples were assayed using Bright-Glo™ Luciferase Assay system (Promega, WI) according to manufacturer’s protocol using Lumat LB 9507 luminometer.

### Adenovirus Constructs and FoxO3a Constructs

For PTEN adenovirus construction, wild type (WT) PTEN cDNA was generated from normal human lung fibroblasts (HLF-210) by RT-PCR. The primers used for wild type PTEN, which span the entire coding region of PTEN are 5′-CTA CTC GAG GCT CCC AGA CAT GAC-3′ and 5′-ACG CTC GAG ATA AAA AAA AAT TCA G-3′. The lipid and protein phosphatase truncated mutant was generated by PCR with primers 5′ -CAG CTCGAGGGACGAACTGGTGTA A-3′ and 5′ -ACG CTC GAG ATA AAA AAA AAT TCAG-3′. The amplified products were cloned into MIGR1-IRES-GFP followed by *SAl*I and *EcoR*I digestion. Wild type PTEN MIGR1-IRES-GFP fragments were then cloned into the adenoviral vector, pAxCAwt (purchased from Dakara Bio. Inc., Japan) and adenovirus titer was measured by plaque method in 0.5% soft agar. The cells were infected with adenoviral vectors at a multiplicity of infection of 1∶20. For the transient transfection assay, plasmids expressing wild type (WT), mutant (S253A) or triple mutant FoxO3a (TM) which three phosphorylation residues recognized by Akt were mutated to alanine were obtained from Addgene. Adenovirus expressing HA-tagged Akt with c-src myristolyation sequence fused in frame to the N terminus (Hyper active Akt), HA-tagged Akt dominant mutant (T308A, S473A) and wild type FoxO3a were purchased from Vector Biolabs (Eagleville, PA). Adenovirus expressing GFP tagged wild type FoxO3a, dominant negative FoxO3a with the deletion of the transactivation domain from the c-terminus and empty vector were also purchased from Vector BioLabs. Control or IPF fibroblasts were infected with adenovirus expressing PTEN, Akt or empty vector in 2 ml of tissue culture dishes. 1 to 2×10^5^ control or IPF fibroblasts per ml of DMEM medium were infected with 1×10^6^ PFU of each adenovirus for assay.

### Immunohistochemistry

Patient specimens were fixed overnight with paraformaldehyde (4%) at RT. The specimens were rinsed in 6% sucrose/0.1 M Dulbeccos PBS (DPBS) at RT, infiltrated with increasing concentrations of Sucrose/DPBS with rotation at 4°C over a 72 h period, and cryoprotected first with 20% sucrose/DPBS overnight, then in a 1∶1 solution of 20% sucrose in DPBS/O.C.T (Sakura Finetek, Torrance, CA), and rotated overnight at 4°C. After cryoembedding in O.C.T., 4 µ sections were cut and placed on silane-coated slides using a Leica 1950 Cryostat. Cryosections were fixed in 100% methanol at −20°C for 10 minutes, air dried and then quenched with 0.3% Hydrogen peroxide in methanol for 15 minutes at RT, and again air dried. They were then rinsed in PBS, placed in a steamer for 10 min with Citrate Buffer for antigen retrieval, and blocked for 30 min with normal serum determined by the host species of the antibody, to block nonspecific binding of secondary antibodies. Endogenous Avidin and Biotin binding sites were blocked by sequential incubation with Avidin/Biotin Blocking Kit (Vector Laboratories, Burlingame, CA). The sections were then incubated overnight at 4°C in their respected concentrations for each antibody, α- smooth muscle actin (1∶100) (Vector Labs), p-FoxO3A (1∶150) (Santa Cruz, Dallas, TX), cleaved caspase-3 (1∶100) and cav-1(1∶1000) (Cell Signal, Danvers, MA) and Fas (1∶100) (Millipore, Temecula, CA). For detection of the primary antibody, the LSAB Method was used. The sections were first rinsed with PBS, then placed in a species specific biotinylated secondary antibody for 30 min (1∶500), and transferred to the HRP-Streptavidin (Vector Laboratories, Burlingame, CA) conjugate solution for 30 min. The enzyme was then visualized with the application of the chromagen substrate 3, 3′ Diaminobenzene and counterstained with Hematoxylin. Control specimens were processed with the identical protocol minus the primary antibody to produce the negative controls.

### Statistical Analysis

Data are expressed as the mean ± S.D. Experiments were performed three times. Paired evaluations were made for experimental and control conditions within each experiment, and significance was determined by Student's t test. Significance level was set at p<0.05.

## Results

### Low FoxO3a Confers IPF Fibroblasts with Resistance to Polymerized Collagen-Mediated Apoptosis

**Figure 1:**
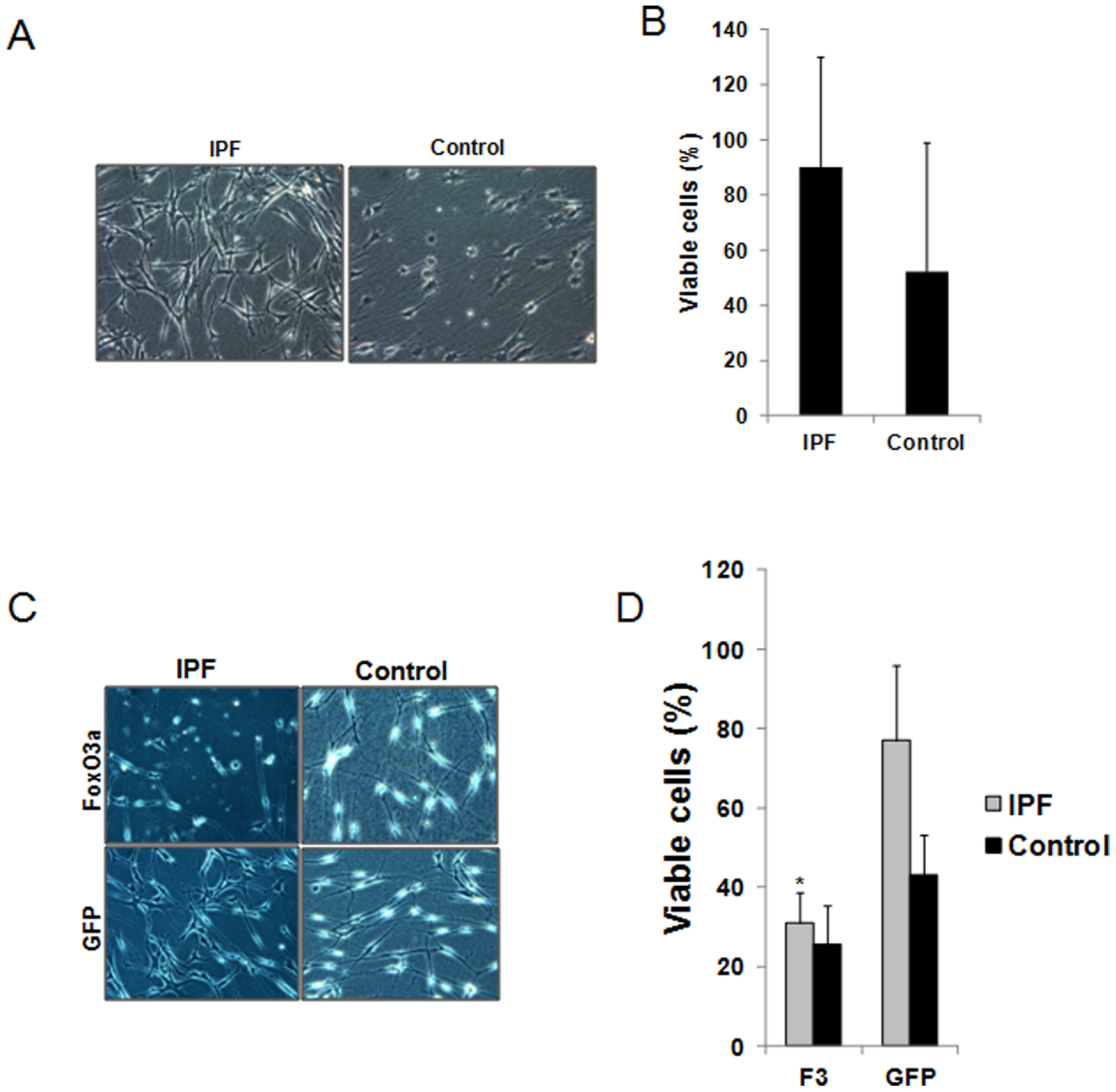
Low FoxO3a confers IPF fibroblasts with resistance to polymerized collagen-mediated apoptosis. **A & B**. Serum starved IPF and control fibroblasts (n = 5, each) were permitted to attach to polymerized collagen for 48 h in the absence of serum and cell morphology (**A**) and cell viability (**B**) were examined. Shown are 50X magnification images of cell morphology. **C & D**. IPF and control fibroblasts infected with adenovirus expressing FoxO3a (F3) or empty vector GFP were cultured on collagen for 48 h in serum free medium and cell morphology (**C**) and cell viability (**D**) were examined. Shown is 50X magnification of cell morphology. Note that forced expression of FoxO3a significantly increased apoptosis in IPF fibroblasts on collagen matrix. *p = 0.003 versus GFP control. The assay was obtained from triplicate.

We have previously found that normal fibroblasts undergo apoptosis during their interaction with and contraction of polymerized collagen matrices [9]. Since IPF is characterized by the proliferation and persistence of fibroblasts within a type I collagen-rich matrix [10], we hypothesized that they may be resistant to polymerized collagen-mediated apoptosis. To examine this possibility, we examined the viability of IPF and control fibroblasts on polymerized collagen matrix under various conditions. When control fibroblasts were cultured on polymerized collagen for 48 h, the cells adopted a rounded morphology (Fig. 1A). In contrast, IPF fibroblasts displayed an elongated fibroblastic morphology on polymerized collagen matrix. Quantification of viability revealed that ∼45% of control fibroblasts were viable vs. ∼85% of IPF fibroblasts (Fig. 1B). This suggests that IPF fibroblasts are resistant to polymerized collagen-mediated apoptosis, compared to control fibroblasts.

Active FoxO3a can promote apoptosis. Since FoxO3a function is abnormally low in IPF fibroblasts, we next examined whether up-regulation of FoxO3a expression in IPF fibroblasts alters their morphology and viability when they interact with polymerized collagen. When FoxO3a was overexpressed in IPF fibroblasts on collagen matrix, they adopted a round appearance and apoptotic cells were readily apparent whereas IPF fibroblasts treated with GFP empty vector maintained their fibroblast morphology (Fig. 1C). The percentage of IPF fibroblasts that were viable markedly decreased to ∼30% when FoxO3a was overexpressed (Fig. 1D). FoxO3a over-expression in control fibroblasts displayed a largely apoptotic morphology on polymerized collagen similar but slightly more pronounced than control cells expressing GFP control. (Fig. 1C) Viability, although minimally altered, remained poor presumably due to high FoxO3a activity at baseline (Fig. 1D).

### FoxO3a Regulates Cav-1 mRNA and Protein Expression

**Figure 2:**
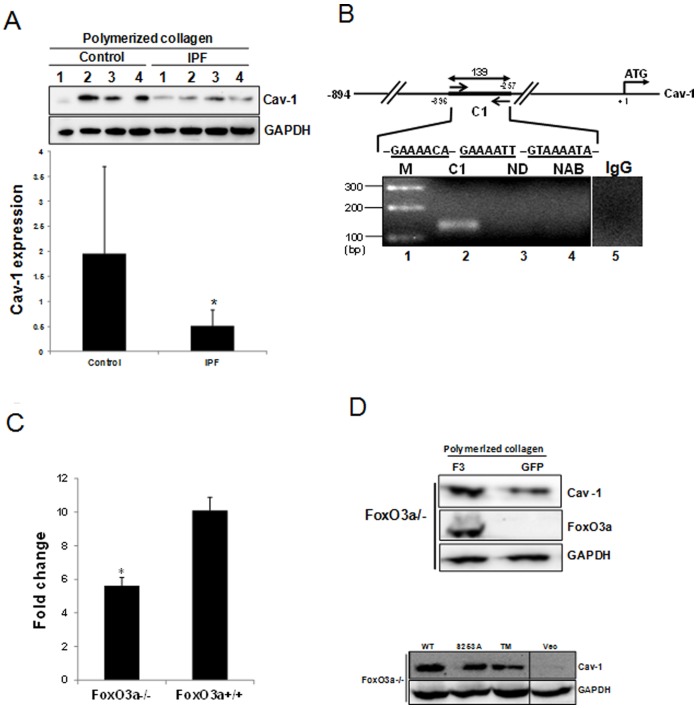
FoxO3a regulates cav-1 mRNA and protein expression. **A**. Upper, shown is a representative Western blot analysis for cav-1 protein expression in control (n = 4) and IPF fibroblasts (n = 4) cultured on type I polymerized collagen matrix in serum free medium for 24 h. Lower, serum starved IPF (n = 8) and control fibroblasts (n = 6) were cultured under the same condition. Shown is cav-1/actin expression ratio of control and IPF fibroblasts on polymerized collagen. *p = 0.02 versus control fibroblasts. **B**. ChIP assay was carried out with primers as described in the Materials and Methods. Putative FoxO3a consensus binding sequences were underlined within C1 region. 1/25 volume (2 µl) of recovered DNA with primers flanking C1 region was used for PCR and 139 bp of ChIP-PCR product was amplified. ChIP-PCR without recovered DNA was carried out using C1 forward and reverse primers as a negative control (ND). ChIP-PCR without FoxO3a antibody using C1 forward and reverse primers was also carried out as an antibody control (NAB). ChIP-PCR from immunoprecipitate with normal rabbit IgG using C1 forward and reverse primers was performed as an IgG control (IgG). Image was obtained from a different gel. M: 1kb DNA ladder. **C**. Quantitative RT-PCR was carried out using primers as described in Materials and Methods. Shown is quantitative RT-PCR normalized to 18S rRNA in FoxO3a−/− and FoxO3a+/+ cells. *p = 0.002. The assay was obtained from triplicate. **D**. Upper panel, FoxO3a −/− cells were infected with adenovirus expressing wild type FoxO3a (F3) or empty vector GFP, and cultured on polymerized collagen for 24 hrs. Cav-1 protein expression was measured by Western analysis. GAPDH was used as a loading control. Lower panel. FoxO3a −/− cells transfected with constructs expressing wild type FoxO3a (WT), or S253A mutant FoxO3a (S253A) or a FoxO3a construct with all Akt phosphorylation residues mutated with alanine (TM) were cultured on polymerized collagen for 24 hrs. Cav-1 protein levels were measured by Western blot analysis. Vec: empty vector. Images were obtained from same Western blot.

Recent studies have demonstrated that caveolin-1 (cav-1) regulates the Fas-mediated apoptotic pathway [36], raising the possibility that FoxO3a may regulate IPF fibroblast viability on polymerized collagen matrices via cav-1. In support of this concept, a prior study suggested that FoxO regulates cav-1 expression [30]. Since both cav-1 expression and FoxO3a activity are low when IPF fibroblasts interact with polymerized collagen [11], [12], we hypothesized that cav-1 expression was suppressed due to inappropriately low FoxO3a activity in IPF fibroblasts on collagen. To test this hypothesis, we first analyzed cav-1 expression in control and IPF fibroblasts. We have previously found that cav-1 is aberrantly low in IPF fibroblasts cultured on collagen [12]. In this study, we further measured fold change of cav-1 protein levels in control and IPF fibroblasts on collagen matrix. Densitometric analysis demonstrated that cav-1 expression was ∼4-fold higher in control fibroblasts compared with that of IPF fibroblasts (Fig. 2A upper and lower panels). These data demonstrate that unlike control fibroblasts, cav-1 expression in response to IPF fibroblast interaction polymerized collagen is aberrantly suppressed.

Prior studies indicate that the cav-1 promoter contains putative FoxO consensus binding sites (5′-GTAAA(C/T)A-3′) [30]–[32], [42]. Recent studies further suggest that FoxO3a can bind to a AT-rich conserved insulin response sequence (IRS) (5′-CAAAA(C/T)A-3′) [33], [39]. Therefore, we examined whether FoxO3a can bind to these potential binding sites and induce cav-1 mRNA expression. We first performed a sequence analysis of the cav-1 promoter region (accession number AF019742) using MATCH program version 1.0. This program is designed for searching potential binding sites for transcription factors (TFBS) in DNA sequences. Our sequence analysis revealed that there are putative FoxO3a consensus binding sequences on the cav-1 promoter region. To confirm that FoxO3a binds to these binding sites of the promoter region of cav-1, we performed a ChIP assay. We designed primers that correspond to the region containing 3 putative FoxO3a binding sites including IRS sequence as shown in Fig. 2B. When PCR was performed, the expected 139 bp PCR product was generated (Fig. 2B lane 2). No PCR product was amplified when PCR was carried out without DNA (ND, lane 3) or with recovered DNA from immunoprecipitate in the absence of FoxO3a antibody, (NAB, lane 4) or with IgG control antibody (lane 5). These data confirm that FoxO3a binds to the cav-1 promoter region of fibroblasts. We next analyzed whether cav-1 expression is transcriptionally regulated by FoxO3a. For this assay, we first performed quantitative RT-PCR normalized to 18S rRNA to precisely measure cav-1 mRNA levels in FoxO3a−/− and FoxO3a+/+ cells. Cav-1 mRNA was ∼40% lower in FoxO3a−/− cells compared to that of cav-1+/+ cells (Fig. 2C). These data clearly demonstrate that the absence of FoxO3a is responsible for low cav-1 mRNA level.

To further verify whether FoxO3a is responsible for low cav-1 protein expression in FoxO3a −/− cells, we re-constituted wild type FoxO3a in FoxO3a−/− cells, and cav-1 expression was measured. Cav-1 protein level was high when wild type FoxO3a was expressed compared to that of cells expressing GFP (Fig. 2D upper panel), showing that FoxO3a increases cav-1 protein expression. To confirm this finding, we next analyzed the effect of over-expression of wild type or mutant FoxO3a constructs that localize to the nucleus and are transcriptionally active on cav-1 protein levels in FoxO3a null cells. Expression of wild type FoxO3a, S253A and TM FoxO3a constructs (three phosphorylation residues recognized by Akt are mutated to alanine to be active) in FoxO3a null cells all increased cav-1 expression compared to empty vector (Fig. 2D lower panel). These data confirm that cav-1 protein levels are regulated by FoxO3a activity. Collectively, these data demonstrate that FoxO3a regulates cav-1 mRNA and protein expression.

**Figure 3:**
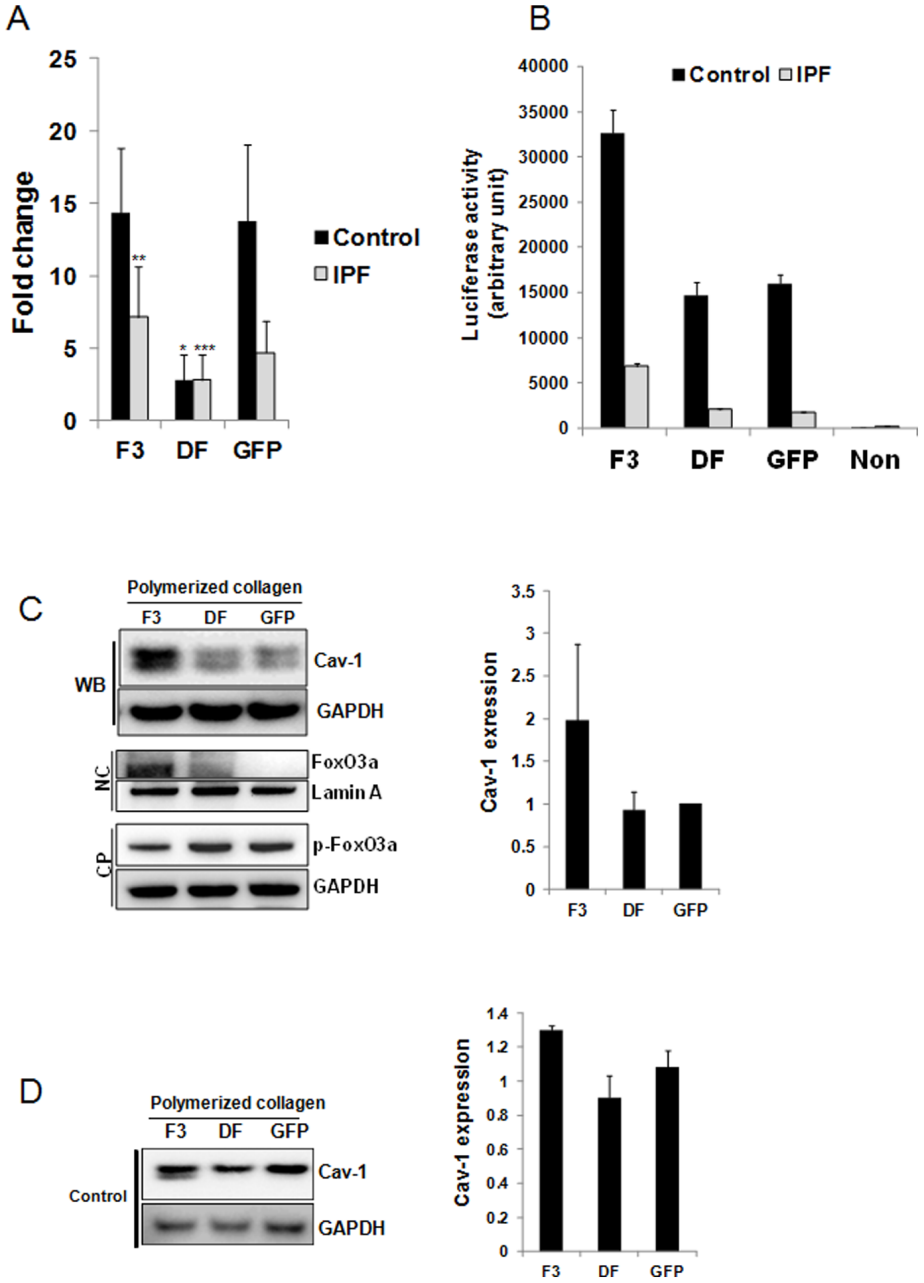
FoxO3a deficiency suppresses cav-1 expression in IPF fibroblast on collagen matrix. **A**. IPF and control fibroblasts infected with adenovirus expressing wild type FoxO3a (F3), dominant negative FoxO3a (DF) or empty vector (GFP) were cultured on polymerized collagen for 24 h in serum free medium. After total RNA was isolated, quantitative RT-PCR was carried out with cav-1 primers as described in the Materials and Methods. Shown is quantitative RT-PCR for cav-1 mRNA levels normalized to 18S rRNA in IPF and control fibroblasts. *p = 0.03 versus GFP in control fibroblasts, **p = 0.02 versus GFP in IPF fibroblasts. ***p = 0.016 versus GFP in IPF fibroblast. The assay was obtained from triplicate from 2 control and 1 IPF fibroblasts. **B**. IPF and control fibroblasts expressing wild type FoxO3a (F3), mutant FoxO3a (DF) or empty vector (GFP) were transfected with a luciferase construct containing consensus Forkhead binding sites (FHRE-Luc). Cells were attached to polymerized collagen in serum free medium for 24 h and luciferase activity was measured as described in the Materials and Methods. Non: cells without transfection of FHRE-Luc construct. The assay was obtained from triplicate. **C**. Left upper panel, shown is representative Western blot analysis (WB) of cav-1 protein expression in IPF fibroblasts expressing wild type (F3), dominant negative FoxO3a (DF) or GFP control and cultured on polymerized collagen for 24 h. Left middle panel, nuclear fraction was isolated from IPF fibroblasts cultured on collagen as described in the Materials and Methods and unphosphorylated FoxO3a protein level was measured. NC : nuclear fraction. Lamin A was used as a loading control. Left lower panel : cytoplasmic fraction was isolated from IPF fibroblasts cultured on collagen and phosphorylated FoxO3a (p-FoxO3a) was measured. CP : cytoplasmic fraction. GAPDH is shown as a loading control. Right panel, densitometric analysis of cav-1/GAPDH expression ratio. **D**. Left panel, control fibroblasts expressing wild type (F3), dominant negative FoxO3a (DF) or GFP control and cultured on polymerized collagen for 24 h and cav-1 protein expression was measured. GAPDH is shown as loading control. Right panel, densitometric analysis of cav-1/GAPDH expression ratio. All blots represent 3 independent assays.

We next sought to examine the possibility that low cav-1 expression in IPF fibroblasts is due to the suppression of cav-1 mRNA transcription as a result of FoxO3a inactivation. To test this, we examined cav-1 mRNA levels in control and IPF fibroblasts expressing wild type FoxO3a, mutant FoxO3a or GFP seeded on polymerized collagen matrices and cav-1 mRNA level was measured using quantitative RT-PCR (Fig. 3A). Cav-1 mRNA level was ∼3 fold higher when empty vector (GFP) was expressed in control fibroblasts compared to that of IPF fibroblasts (Fig. 3A). This finding demonstrates that cav-1 mRNA expression remains low when IPF fibroblasts are cultured on polymerized collagen. However, when mutant FoxO3a was expressed in control fibroblasts, cav-1 mRNA expression was significantly decreased (Fig. 3A). In contrast, when wild type FoxO3a was expressed in control fibroblasts, cav-1 mRNA was not significantly altered as compared with the level of empty vector GFP expression presumably due to pre-existing high FoxO3a activity. However, unlike control fibroblasts, we found that the cav-1 mRNA level was increased in the presence of wild type FoxO3a in IPF fibroblasts (Fig. 3A) whereas cav-1mRNA was moderately decreased when dominant negative FoxO3a was expressed. These results demonstrate that low cav-1 transcriptional level is due to suppression of FoxO3a activity in IPF fibroblasts on collagen. To confirm our finding that cav-1 level is transcriptionally low due to FoxO3a suppression in IPF fibroblasts on polymerized collagen, we utilized a reporter construct that contains consensus FoxO3a binding sites within the promoter of luciferase gene (FHRE-Luc) [46], and luciferase activity was measured in IPF and control fibroblasts expressing wild type FoxO3a, dominant negative FoxO3a or GFP. Luciferase activity was significantly higher (∼6 fold) when control fibroblasts were cultured on polymerized collagen compared to that of IPF fibroblasts (Fig. 3B, GFP). This finding confirms that FoxO3a-dependent transcriptional activity is suppressed in IPF fibroblasts during their interaction with type I collagen. However, when wild type FoxO3a was expressed in IPF fibroblasts, luciferase activity was increased ∼3.2 fold. In contrast, dominant negative FoxO3a had no effect on luciferase activity. Luciferase activity was also increased (∼2 fold) when wild type FoxO3a was expressed in control fibroblasts while dominant negative FoxO3a marginally decreased luciferase activity. Collectively, these data demonstrate that FoxO3a activity is aberrantly low in response to IPF fibroblast interaction with collagen matrix, thereby suppressing FoxO3a-dependent transcriptional activation of cav-1 on polymerized collagen.

We next examined the effect of modulation of FoxO3a expression on cav-1 protein levels in IPF fibroblasts. We over-expressed wild type FoxO3a (F3), dominant negative FoxO3a (DF) or GFP in IPF fibroblasts cultured on polymerized collagen and measured cav-1 levels. Cav-1 protein expression increased in IPF fibroblasts in which wild type FoxO3a was over-expressed (Fig. 3C upper and right panels). Dominant negative FoxO3a had little effect on cav-1 level presumably due to pre-existing low FoxO3a activity in IPF fibroblasts when cultured on polymerized collagen. We have previously demonstrated an absence of non-phosphorylated FoxO3 (active FoxO3a) in the nucleus of IPF fibroblasts when cultured on polymerized collagen [11]. Instead, we have found that p-FoxO3a (inactive FoxO3a) is located in the cytoplasm of IPF fibroblasts. Since wild type FoxO3a transcriptionally increases cav-1, we next examined whether the over-expression of wild type FoxO3a promotes the localization of non-phosphorylated FoxO3a in the nucleus of IPF fibroblasts. For this assay, nuclear and cytoplasmic fractions were isolated from IPF fibroblasts cultured on collagen, and active FoxO3a and inactive FoxO3a levels were measured. We found that FoxO3a was predominantly located in the nuclear fraction when wild type FoxO3a was expressed while FoxO3a levels were low or absent when dominant negative or empty vector were expressed (Fig. 3C, NC, middle panel). In contrast, p-FoxO3a level was low in the cytoplasm when wild type FoxO3a was expressed while increased p-FoxO3a levels were found in the cytoplasm when dominant negative or GFP were expressed (Fig. 3C, CP, lower panel). Taken together, our results demonstrate that forced activation of FoxO3a increases cav-1 expression via enhanced localization of active FoxO3a in the nucleus.

We further examined cav-1 protein levels in control fibroblasts on polymerized collagen. Unlike IPF fibroblasts, wild type FoxO3a did not significantly alter cav-l protein expression compared with that of empty vector expressed (Fig. 3D left and right panels). However, when dominant negative FoxO3a was expressed, cav-1 expression modestly decreased. Taken together, these results demonstrate that FoxO3a regulates cav-1 expression, and aberrantly low FoxO3a is responsible for the suppression of cav-1 expression in IPF fibroblasts on polymerized collagen.

### Aberrant Function of the PTEN/Akt Axis Inactivates FoxO3a and Suppresses Cav-1 Expression

**Figure 4:**
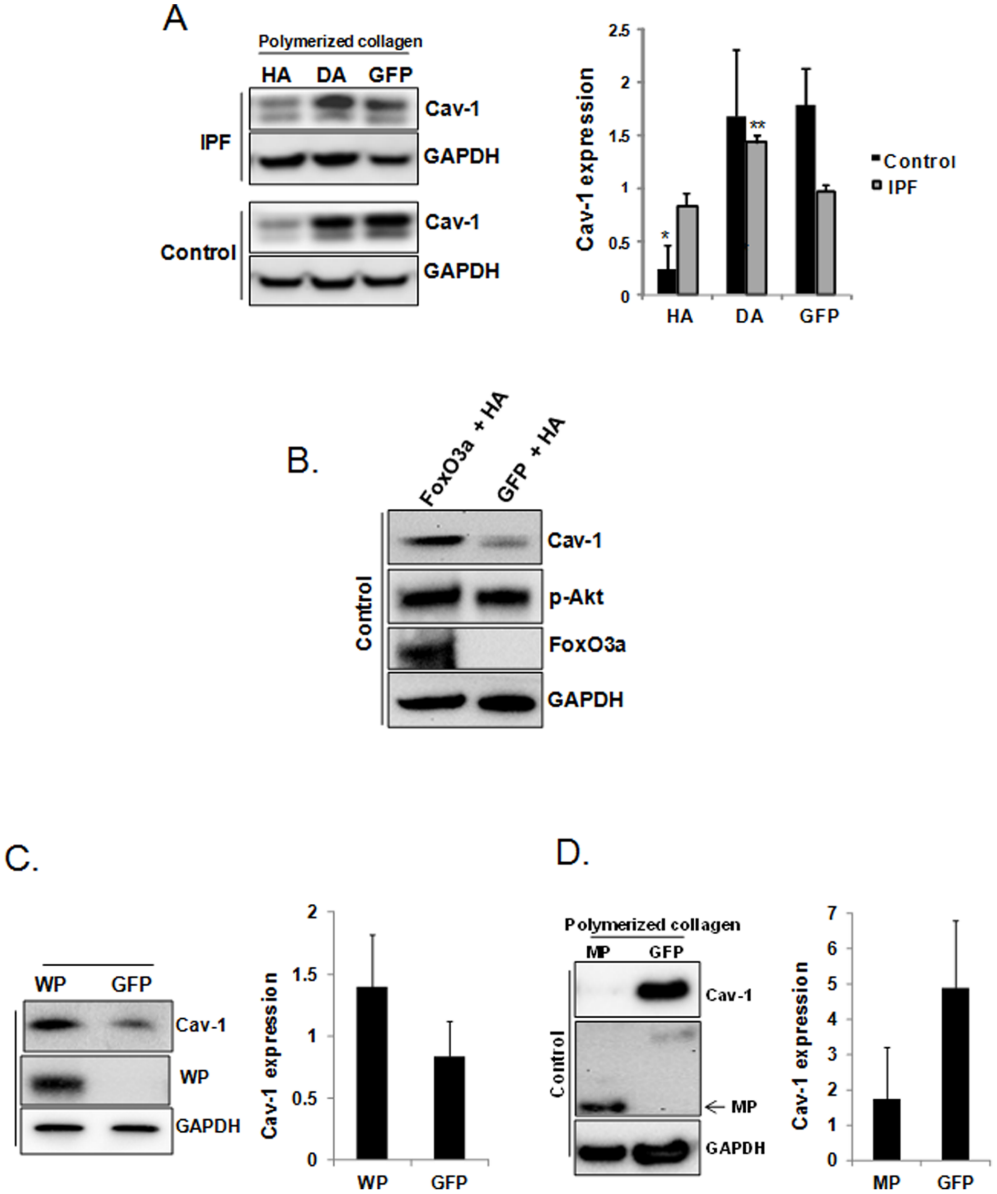
Aberrant function of the PTEN/Akt axis inactivates FoxO3a and suppresses cav-1 expression. **A**. Left panel. IPF and control fibroblasts infected with adenovirus expressing hyperactive Akt (HA), dominant negative Akt (DA) or empty vector GFP were cultured on polymerized collagen and cav-1 protein expression was examined by Western blot analysis. GAPDH is shown as a loading control. Right panel, cav-1/GAPDH expression ratio was quantified by densitometry. *p = 0.017, **p = 0.008 versus GFP control. **B**. Control fibroblasts infected with adenovirus co-expressing either wild type FoxO3a & hyperactive Akt or empty GFP & hyperactive Akt were cultured on polymerized collagen in serum free medium for 24 h, and Western analysis was carried out to measure cav-1, p-Akt, FoxO3a and GAPDH levels. Note that cav-1 level is increased when FoxO3a and hyperactive Akt were co-expressed. **C**. Left, IPF fibroblasts infected with adenovirus expressing wild type PTEN (WP) or empty vector GFP were cultured on polymerized collagen for 24 h and cav-1 protein expression was examined by Western blot analysis. GAPDH is shown as loading control. Right, cav-1/GAPDH levels were quantified by densitometry. **D**. Left, control fibroblasts infected with adenovirus expressing mutant PTEN (MP) or empty vector GFP were cultured on polymerized collagen for 24 h and cav-1 protein levels were examined by Western blot analysis. GAPDH is shown as loading control. Right, cav-1/GAPDH levels were quantified by densitometry. All blots represent at least 3 independent assays.

Our previous work has demonstrated that when IPF fibroblasts interact with polymerized collagen PTEN function is aberrantly low which causes inappropriately high Akt activity and subsequent phosphorylation and inactivation of FoxO3a [11]. Therefore, we next sought to examine the role of Akt in regulating cav-1 expression in IPF fibroblasts. In order to test this, we over-expressed hyperactive (HA), dominant negative Akt (DA) or empty vector (GFP) in control and IPF fibroblasts and cav-1 proteins levels were measured. Cav-1 expression increased when dominant negative Akt was expressed in IPF fibroblasts whereas dominant negative Akt had very little effect on cav-1 expression in control fibroblasts (Fig. 4A lane 2 & right panels). This supports the concept that inhibition of the inappropriately high Akt activity in IPF fibroblasts activates FoxO3a thereby increasing cav-1 expression. This finding also indicates that Akt activity is physiologically suppressed during control fibroblast interaction with polymerized collagen and that further inhibition of Akt does not significantly suppress cav-1 expression. In support of this we found that hyperactive Akt profoundly decreased cav-1 expression in control fibroblasts (Fig. 4A lane 1 lower & right panels). In contrast, hyperactive Akt only slightly altered cav-1 expression in IPF fibroblasts interacting with polymerized collagen, presumably because of pre-existing aberrantly high Akt activity in these cells. To further demonstrate that Akt suppresses cav-1 via FoxO3a, control fibroblasts were infected with adenovirus expressing hyperactive Akt (HA), FoxO3a or GFP, and cav-1 levels were measured on collagen. Cav-1 level was low when HA and GFP were co-expressed (Fig. 4B). In contrast, cav-1 level was high when FoxO3a was co-expressed with HA. These results demonstrate that cav-1 is regulated by Akt/FoxO3a axis.

We have previously shown that PTEN function is inappropriately low in response to IPF fibroblast attachment to polymerized collagen whereas PTEN expression is physiologically high when control fibroblasts are cultured on polymerized collagen. Because low PTEN results in inappropriately high Akt activity in IPF fibroblasts, we sought to examine the role of PTEN on aberrant cav-1 expression. When wild type PTEN was over-expressed in IPF fibroblasts, cav-1 protein levels increased (Fig. 4C left and right panels). In contrast, when mutant PTEN was over-expressed in control fibroblasts, cav-1 protein expression was reduced (Fig. 4D left and right panels). Collectively, our data strongly support the concept that during IPF fibroblast interaction with polymerized collagen, aberrantly activated Akt due to low PTEN function inhibits FoxO3a transcriptional activity thereby suppressing cav-1 expression.

### Low FoxO3a and Cav-1 Function Confers IPF Fibroblasts with an Apoptotic-Resistant Phenotype via Fas Suppression

**Figure 5:**
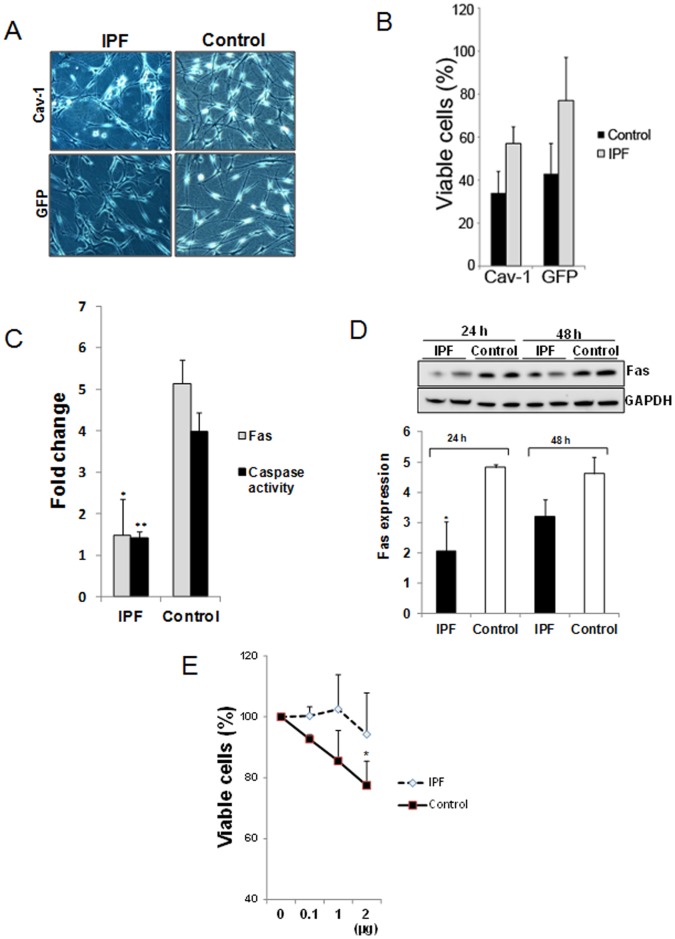
Low FoxO3a and cav-1 function confers IPF fibroblasts with an apoptotic-resistant phenotype via Fas suppression. A & B. IPF and control fibroblasts over-expressing cav-1 or empty vector GFP were cultured on collagen for 48 h in serum free medium and cell morphology (A) and cell viability (B) were examined. Shown is 50X magnification of cell morphology. C. Serum starved IPF and control fibroblasts were cultured on polymerized collagen for 24 h in the absence of serum, and Fas/GAPDH protein expression level and caspase-3/7 activity were measured as described in the Materials and Methods (n = 4 lines each). *p = 0.02, **p = 0.015 versus control fibroblasts. D. Upper panel. IPF and control fibroblasts were cultured on collagen as a function of time, and Fas and GAPDH protein expression was measured. Lower panel. Shown is the Fas/GAPDH protein expression ratio in IPF and control fibroblasts cultured on polymerized collagen as a function of time (n = 2 lines each). E. 2×10^5^ control and IPF fibroblasts cultured on polymerized collagen in the absence of serum were ligated with 0–2 µg of human Fas activating antibody for 18 h. Viable cells were measured as described in the Materials and Methods. Shown are the % viable cells exposed to the various concentrations of Fas activating antibody compared with that of untreated cells. *p = 0.016 versus non treated cells. Results were obtained from triplicates.

Given the fact that FoxO3a increases cav-1 expression and FoxO3a overexpression promotes IPF fibroblast apoptosis on collagen, we next examined the effect of cav-1 overexpression on IPF fibroblast morphology and viability when cultured on polymerized collagen. Interestingly, over-expression of cav-1 altered IPF fibroblast morphology to a more rounded appearance but to a lesser extent compared to FoxO3a over-expressing cells. Consistent with this, overexpression of cav-1 also decreased the percentage of viable IPF cells but to a lesser magnitude than over-expression of FoxO3a (Fig. 5A and B). In contrast, there was a marginal decrease in viable cells when cav-1 was over-expressed in control fibroblasts on collagen.

We next sought to investigate the mechanism by which FoxO3a and cav-1 reduce IPF fibroblast viability. Prior work indicates that low Fas expression protects IPF fibroblasts from Fas mediated apoptosis, while another study found that cav-1 can regulate Fas function [36], [37], suggesting that IPF fibroblast viability in response to interaction with polymerized collagen may be dependent on Fas expression. We first examined Fas levels in IPF fibroblasts cultured on collagen matrix. Similar to cav-1 expression, Fas expression was also lower in IPF fibroblasts cultured on polymerized collagen compared to control fibroblasts (Fig. 5C). We further measured Fas protein levels in IPF and control fibroblasts on collagen as a function of time. Although Fas expression increased modestly in IPF fibroblasts as a function of time, Fas expression remained lower in IPF fibroblasts compared to control fibroblasts (Fig. 5D). Since Fas promotes fibroblast apoptosis by activating caspase-3 and 7 via caspase-8 [38], we next measured caspase 3/7 activity in IPF and control fibroblasts on polymerized collagen. Caspase 3/7 activity was much lower in IPF fibroblasts compared to control fibroblasts (Fig. 5C). To confirm that low Fas protein expression is responsible for IPF fibroblast resistance to collagen-mediated apoptosis, control and IPF fibroblasts were ligated with various doses of Fas activating human antibody, and cell viability measured. The viability of control fibroblasts decreased in a dose-dependent fashion (Fig. 5E). In contrast, IPF fibroblast viability on polymerized collagen was not significantly altered by Fas activating antibody. These results demonstrate that Fas expression is low in IPF fibroblasts interacting with type I collagen and suggest that low Fas expression confers IPF fibroblasts with resistance to polymerized collagen-mediated apoptosis.

### Forced Expression of FoxO3a Increases Fas Expression via Cav-1 in IPF Fibroblasts and Promotes Apoptosis on Polymerized Collagen

**Figure 6:**
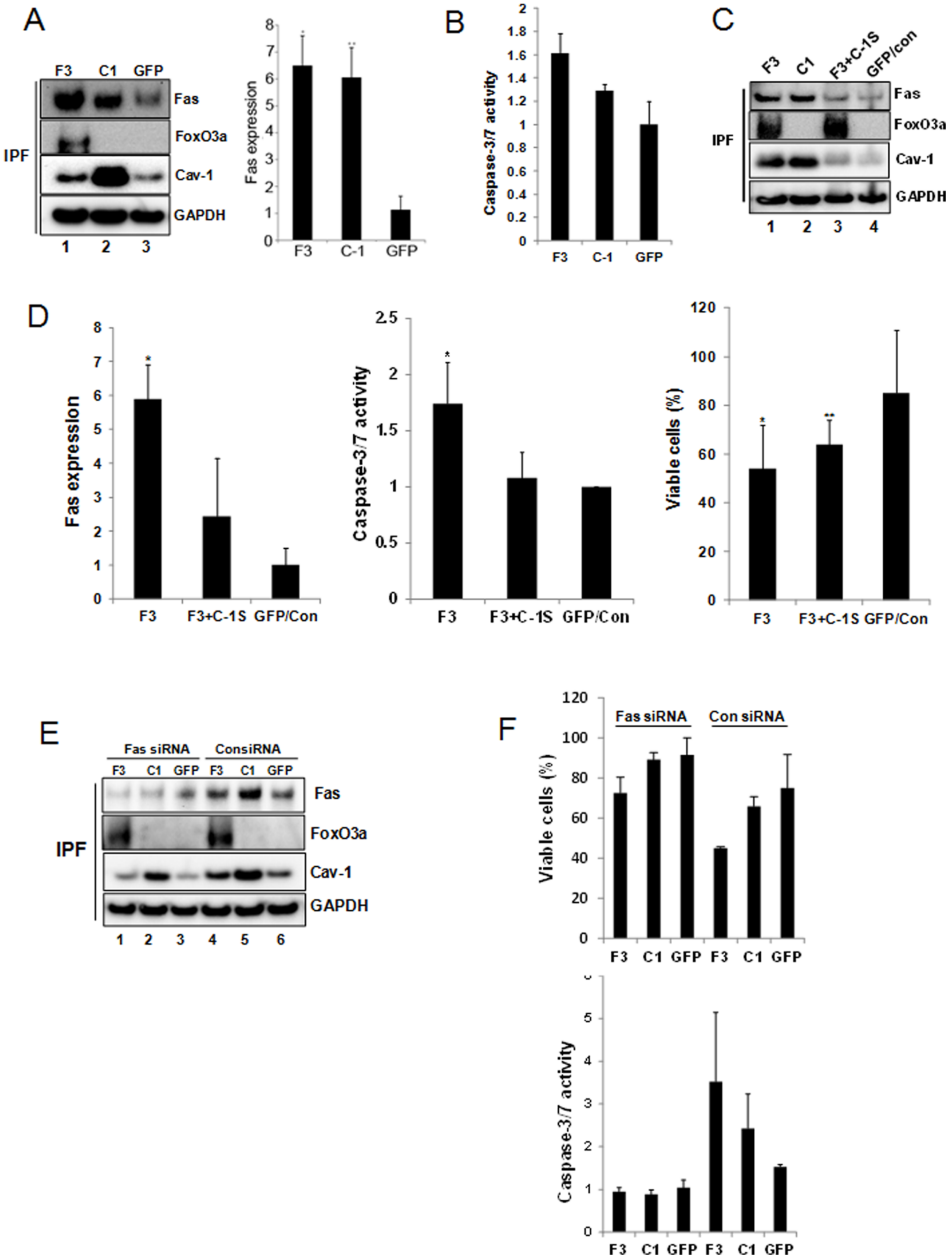
Forced expression of FoxO3a increases Fas expression via cav-1 in IPF fibroblasts and promotes apoptosis on polymerized collagen matrix. A. IPF fibroblasts infected with adenovirus over-expressing FoxO3a (F3), cav-1 (C-1) or empty vector GFP were cultured on polymerized collagen for 48 h in serum free medium. Left panel, Fas, FoxO3a, cav-1 and GAPDH expression were measured by Western blot analysis. Three independent assays are represented. Right panel. Fas/GAPDH protein expression ratio was quantified. *p = 0.02 versus GFP. **p = 0.05 versus GFP. B. IPF fibroblasts infected with adenovirus over-expressing FoxO3a (F3), cav-1 (C-1) or empty vector GFP were cultured on polymerized collagen for 48 h and caspase-3/7 activity was measured. Results were obtained from triplicates. C. IPF fibroblasts infected with adenovirus over-expressing FoxO3a (F3), cav-1 (C1), FoxO3a in the presence of 10 nM of Cav-1 siRNA (F3+C-1S), or empty vector GFP with 10 nM control siRNA (GFP/Con) were cultured on polymerized collagen for 48 hrs. Fas, FoxO3a, cav-1 and GAPDH expression were examined by Western blot analysis. Blot represents 3 independent assays. D. IPF fibroblasts infected with adenovirus expressing FoxO3a alone (F3) or IPF fibroblasts over-expressing FoxO3a in the presence of 10 nM of Cav-1 siRNA (F3+C-1S) or GFP/Control siRNA (GFP/Con) were cultured on polymerized collagen for 48 hrs. Left panel, Fas expression was quantified. *p = 0.03 versus GFP/Con. Results were obtained from 3 independent assays. Middle panel, Caspase-3/7 activity was measured. *p = 0.01 versus GFP/Con. Results were obtained from triplicates. Right panel, viable cells were quantified. *p = 0.01, **p = 0.05 versus GFP/Con, respectively. Results were obtained from quadruplicates. E–F. 2×10^5^ IPF fibroblasts infected with adenovirus over-expressing FoxO3a (F3), cav-1 (C1) or empty vector GFP were cultured on polymerized collagen in the presence of 10 nM Fas or control siRNA for 48 h in serum free medium. Fas, FoxO3a, cav-1 and GAPDH expression were measured by Western blot analysis (E). Cell viability (F, upper panel) and caspase-3/7 activity (F, lower panel) were measured as described in Materials and Methods. Results were obtained from triplicates.

Since FoxO3a can induce cav-1 expression and cav-1 is known to regulate Fas-dependent apoptosis pathway, this suggested the possibility that FoxO3a may regulate IPF fibroblast viability via modulating cav-1 and Fas expression. In order to address this, we first over-expressed FoxO3a or cav-1 in IPF fibroblasts and Fas expression was measured. FoxO3a or cav-1 over-expression significantly increased Fas protein expression (Fig. 6A left panel, lane 1 and 2). Densitometry analysis indicated that Fas protein levels were increased ∼6 fold when IPF cells were infected with adenovirus expressing either FoxO3a or cav-1 (Fig. 6A right panel). When we examined the effect of up-regulation of FoxO3a or cav-1 on caspase-3/7 activity in IPF fibroblasts cultured on polymerized collagen, caspase-3/7 activity was increased by 60% and 30%, respectively (Fig. 6B). Together, these data demonstrate that forced expression of FoxO3a and cav-1 in IPF fibroblasts on polymerized type I collagen matrix increases Fas expression and caspase activity.

To determine whether FoxO3a regulates IPF fibroblast viability via a cav-1 dependent mechanism, we next measured Fas protein expression, caspase-3/7 activity, and cell viability in IPF fibroblasts expressing FoxO3a in the presence or absence of cav-1 siRNA. When FoxO3a or cav-1 was over-expressed, Fas expression was highly increased (Fig. 6C lanes 1 and 2, Fig. 6D left panel). However, when cav-1 was silenced by cav-1 siRNA in IPF fibroblasts over-expressing FoxO3a, Fas expression remained low (Fig. 6C lane 3 and Fig. 6D left panel). Furthermore, caspase-3/7 activity was significantly increased in IPF fibroblasts in which FoxO3a was over-expressed, while caspase-3/7 activity was only marginally increased when cav-1 was knocked-down in IPF fibroblasts over-expressing FoxO3a (Fig. 6D middle panel). Consistent with these findings, when FoxO3a was over-expressed, the percentage of IPF fibroblasts undergoing apoptosis was ∼50% compared to ∼17% apoptosis in IPF fibroblasts expressing empty vector (Fig. 6D right panel). However, when cav-1 was silenced by cav-1 siRNA in IPF cells over-expressing FoxO3a, the percentage of cells undergoing apoptosis was ∼35%, indicating that knockdown of cav-1 attenuates FoxO3a-induced apoptosis (Fig. 6D right panel). Taken together, these results demonstrate that FoxO3a regulates IPF fibroblast viability at least, in part, via cav-1 and that abnormally low FoxO3a protects IPF fibroblasts from polymerized collagen induced apoptosis via suppressing cav-1/Fas-dependent pathway.

We next sought to determine whether suppression of Fas expression would protect IPF fibroblasts overexpressing FoxO3a or cav-1 from collagen matrix-induced apoptosis. Knockdown of Fas in IPF cells over-expressing FoxO3a or cav-1 reduced Fas protein levels (Fig. 6E lanes 1 & 2) and significantly decreased IPF fibroblast apoptosis compared to IPF fibroblasts overexpressing FoxO3a or cav-1 but transfected with control siRNA (Fig. 6F upper panel). Consistent with this, caspase-3/7 activity decreased in IPF fibroblasts expressing FoxO3a or cav-1 in the presence of Fas siRNA compared to cells treated with control siRNA (Fig. 6F lower panel). Collectively these data strongly suggest that suppression of Fas expression due to low FoxO3a/cav-1 function is an important mechanism that confers IPF fibroblasts with resistance to polymerized collagen-induced apoptosis.

### Fibroblasts within the Fibroblastic Foci of IPF Patient Lung Specimens Express Inactive FoxO3a and α-smooth Muscle Actin but not Cav-1 and Cleaved Caspase-3

**Figure 7:**
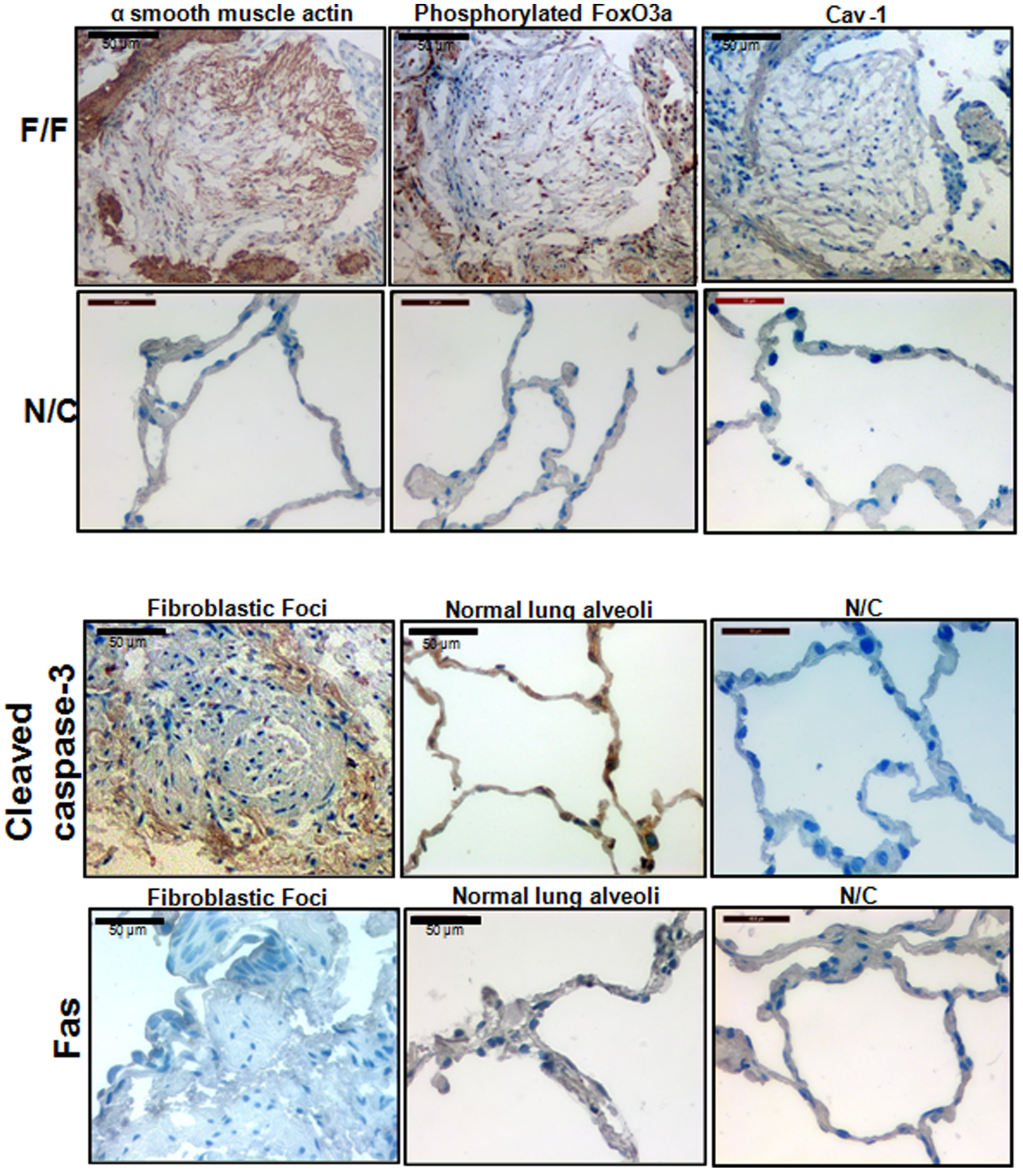
Fibroblasts within the fibroblastic foci of IPF patient lung specimens express inactive FoxO3a and α-smooth muscle actin but not cav-1 and cleaved caspase-3. Upper upper panel, frozen serial sections of IPF patient lung specimens (F/F: fibroblastic foci) were immunostained with anti-α smooth muscle actin antibody (upper left), phosphorylated FoxO3a (inactive FoxO3a, upper middle), or anti-cav-1 (upper right), and IHC was carried out as described in the Materials and Methods (n = 3). Upper lower panel, shown are IHC images obtained from control tissue without primary antibodies as negative controls (N/C). Lower upper panel. IHC analysis of IPF (left) and control (middle) lung tissue was performed using anti-cleaved caspase-3 antibodies. N/C : IHC images obtained from control tissue without cleaved caspase-3 antibody as a negative control. Lower lower panel. Fas expression was measured from frozen sections of IPF (left) and control (middle) lung specimens. N/C : IHC images obtained from control tissue without Fas antibody as a negative control. Note that cav-1, cleaved caspase-3 and Fas expression were absent or very low in cells within the fibroblastic foci.

We analyzed lung pathological specimens from IPF patients to determine the expression of α-smooth muscle actin, cav-1, inactive FoxO3a, cleaved caspease-3, and Fas. Consistent with our previously published results [11], [12], cells within the IPF fibroblast focus were highly immunogenic to phosphorylated FoxO3a (inactive FoxO3a) and α-smooth muscle actin (Fig. 7 upper left and middle, respectively). However, cav-1 and cleaved caspase-3 expression was very low and Fas expression was nearly absent in cells within IPF fibroblastic foci (Fig. 7). Cleaved caspase-3 and Fas immunoreactivity was detected in only occasional cells in the alveoli of control lung tissue (Fig. 7 lower middle panels). Our morphological findings are consistent with the concept that reduced FoxO3a activity facilitates the persistence of IPF fibroblasts within the collagen-rich matrix via suppressing cav-1/Fas expression.

## Discussion

IPF is characterized by relentless progression of fibrosis where fibroblasts proliferate and deposit type I collagen within alveolar structures resulting in scarred nonfunctional airspaces. We have found that IPF fibroblasts display a distinct pathologic phenotype in response to interaction with a collagen rich matrix. The primary reasons for investigating IPF fibroblast function on polymerized type I collagen matrices are two-fold. First, 3-D collagen matrices are a physiologically relevant matrix. IPF fibroblasts reside in a type I collagen rich microenvironment. Second, IPF fibroblasts display an apoptotic-resistant phenotype when cultured on 3-D collagen matrices compared to control fibroblasts. This difference in viability is manifested on collagen and not on tissue culture plastic. The current study further defines the mechanism for this apoptotic-resistant phenotype displayed by IPF fibroblasts on collagen. In IPF fibroblasts, altered integrin function results in inappropriately low PTEN activity, which causes aberrant activation of the PI3K/Akt signal pathway and facilitates their proliferation on collagen. We have previously shown that the aberrantly activated PI3K/Akt pathway phosphorylates and inactivates the FoxO3a transcription factor. FoxO3a is a powerful regulator of the cell cycle and cell viability and when active it inhibits proliferation and promotes apoptosis. Our prior work indicates that normal fibroblasts undergo apoptosis upon contraction of polymerized collagen and is a physiologic mechanism for the elimination of cells during tissue repair. However, in IPF, fibroblasts persist and proliferate. Here we demonstrate that inactivation of FoxO3a in IPF fibroblasts confers these cells with resistance to polymerized collagen-mediated apoptosis and involves a cav-1/Fas-dependent mechanism.

Cav-1 deregulation has been linked to several human diseases [26]–[29]. A direct link between cav-1 alteration and tumorigenesis has been previously demonstrated. Cav-1 absence sensitizes mouse skin to carcinogen induced epidermal hyperplasia and tumor formation [34]. Deletion of caveolin-1 also protects against hyperoxia-induced apoptosis [35]. Experimental evidence also suggests that alterations of cav-1 function can be responsible for ECM production in fibroblasts and abnormal cav-1 function has been linked to lung fibrosis [12], [26]. A prior study suggested that FoxO transcription factors regulate cav-1 expression [30]. Since we have previously found that FoxO3a is inactivated in IPF fibroblasts interacting with type I collagen [11], we hypothesized that aberrantly low FoxO3a activity may be responsible for cav-1 suppression. Sequence analysis revealed that there are putative FoxO consensus binding sites and insulin response sequence (IRS) on the cav-1 promoter regions. Here we demonstrate that FoxO3a binds to the cav-1 promoter and quantitative RT-PCR assay further demonstrated that cav-1 mRNA is low in IPF and FoxO3a−/− cells. Our luciferase assay using FHRE-Luc construct also demonstrate that FoxO3a-dependent transcriptional activity is suppressed in IPF fibroblasts. These data support the concept that pathological inactivation of FoxO3a in IPF fibroblasts suppresses cav-1 expression and contributes to IPF progression.

During physiologic tissue repair, fibroblasts are removed by apoptosis. Because IPF is characterized by the persistence of fibroblasts within a collagen rich matrix, this suggested that these cells may be resistant to collagen-matrix induced apoptosis. We have shown that the mechanism involves collagen contraction-mediated activation of PTEN and inhibition of Akt. Our results using hyperactive or dominant negative Akt constructs demonstrate that Akt activation is a crucial step that confers IPF fibroblasts with an apoptotic-resistant phenotype during their interaction with polymerized type I collagen. Prior studies also showed that Akt activation protect fibroblasts from apoptosis inducing stimuli [40]–[42]. Akt is known to regulate many its down-stream target proteins and among them, FoxO3a has been implicated in longevity to tumor suppression [43]-[46]. Previous studies have demonstrated in important functional role for FoxO3a in suppressing cell proliferation and promoting apoptosis by transcriptionally regulating FoxO3a target genes [42]–[44], [46]. Thus, available data support our findings indicating a direct link between the activation of pro-survival signaling pathways by Akt and an anti-apoptotic phenotype in IPF fibroblasts.

Increased cell surface Fas expression is necessary to sensitize lung fibroblasts to Fas ligation induced apoptosis and Fas expression is minimal in (myo)fibrolasts in the fibroblastic foci [37]. Since FoxO3a and cav-1 can regulate Fas levels, this suggested to us the possibility that in IPF, inactivated FoxO3a decreases cav-l levels, which results in low Fas expression and resistance to apoptosis on collagen matrix. Indeed, we demonstrate that overexpression of either FoxO3a or cav-1 in IPF fibroblasts increases Fas expression and promotes activation of caspase 3/7 activity and induction of apoptosis on polymerized collagen. We also demonstrate that when Fas is silenced in IPF fibroblasts in which FoxO3a or cav-1 is over-expressed, the result is the protection of IPF fibroblasts from collagen matrix driven apoptosis. This indicates that low Fas expression in fibroblasts confers the cells with resistance to apoptosis in response to their interaction with collagen. Since FoxO3a can regulate cav-1 levels and cav-1 can regulate Fas expression, we explored whether FoxO3a regulates IPF fibroblast viability via cav-1/Fas dependent mechanism. We demonstrate that cav-1 over-expression induces IPF fibroblast apoptosis on collagen matrix by up-regulating Fas and caspase activity. Thus these results strongly suggest that alteration of FoxO3a promotes, at least in part, apoptosis via the cav-1/Fas/caspase-3/7. However, interestingly, FoxO3a over-expression in the presence of cav-1 siRNA attenuated but did not completely abrogate IPF fibroblast apoptosis compared with FoxO3a in the absence of cav-1 siRNA. This finding indicates that FoxO3a promotes cell death via cav-1-dependent and independent mechanisms. In fact, studies have shown that FoxO3a promotes cell apoptosis by up-regulating several apoptosis inducing proteins [22]–[24]. Thus it is feasible that when control fibroblasts attach to polymerized collagen, elevated FoxO3a activity increases apoptosis by enhancing multiple FoxO3a target proteins including cav-1.

Although we found that IPF fibroblasts display an apoptotic resistant phenotype on type I polymerized collagen, it is possible that the pepsin extracted collagen that we used for our studies may not fully simulate the polymerized cross-linked collagen that exists in the *in vivo* cell environment. Pepsin extracted process removes nonhelical telopeptides situated at the N- and C-terminal ends of native collagen molecules [50]. Telopeptides play important roles in fibrillogenesis and support the intermolecular covalent cross-links necessary for stabilizing gel architecture [51], [52]. Therefore the structural integrity imparted by reconstituted acid extracted type I collagen, which maintains telopeptides, might be a better model compared to that of pepsin extracted collagen. Nevertheless, a prior study has demonstrated that telopeptides are not essential for collagen fibril formation [53]. Kuznetsova et al. found that complete removal of telopeptides had no significant effect on the fiber forming competency of collagen. Furthermore, electron microscopy and mechanical analysis showed that pepsin extracted collagen I matrix was more fibrillar than Matrigel, with larger inter-fiber distance and higher stiffness [54]. Like control fibroblasts on polymerized collagen, after 24 h in culture, the somata of SH-SY5Ycells cultured in 3D matrices were globular in appearance, whereas cells on 2D substrates exhibited a flatter, more spread morphology. Importantly, we have demonstrated that IPF cells maintain their fibroblastic morphology and viability on pepsin extracted collagen matrix, whereas control fibroblasts are highly susceptible for matrix driven cell death. These data indicate that the 3D collagen matrices utilized in our study provide a matrix environment that distinguishes IPF fibroblasts from their normal counterparts.

Although we focused on the role of FoxO3 in regulating IPF fibroblast function, FoxO3a is also thought to be involved in aging. Since IPF is a disease of older individuals with a mean age of ∼65 years [47], this suggests that IPF is an age-dependent disease. Interestingly, FoxO3a has been linked to aging and longevity. A recent study conducted with Flachsbart et al. found that certain FoxO3a variants are very common in patients >90 years old, and they were even more common in 100 year old individuals [48]. The FoxO family is thought to be involved in maintaining cellular homeostasis during aging. From this perspective, stress resistance is known to be associated with longevity, and activation of the FoxO3a gene is associated with response to stress. Recent studies found that FoxO3a detoxifies ROS by utilizing MnSOD and catalase [49]. Thus FoxO3a’s function in protecting cells may be an important defense mechanism against harmful environmental stress and aging process. Since there is a high incidence of IPF in older individuals, and FoxO3a activity is pathologically low in IPF fibroblasts, we propose that inactivation of FoxO3a may promote IPF progression. Optimum activation of FoxO3a may be crucial for preventing or delaying the onset of IPF. Since we are interested in identifying novel therapeutic approaches to prevent or limit the progression of IPF, re-activation of FoxO3a or inhibition of the FoxO3a inactivation may provide an approach not only for slowing IPF disease progression but also for longevity.

**Figure 8:**
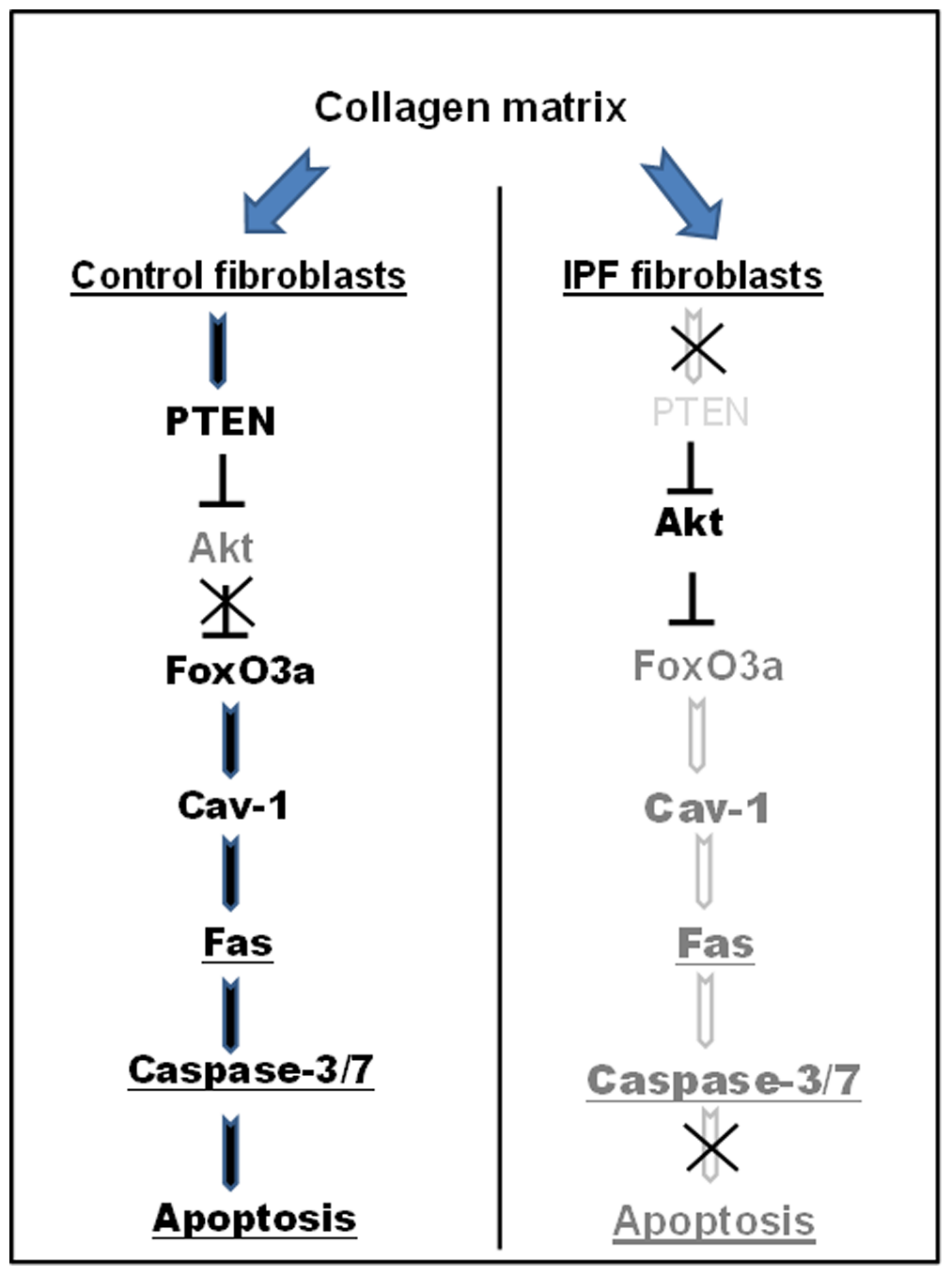
Schematic illustrating the mechanism by which FoxO3a deficiency confers IPF fibroblasts with resistance to polymerized collagen-mediated apoptosis. When control fibroblasts are cultured on polymerized collagen, Akt activity is suppressed by elevated PTEN activity, resulting in an increase in FoxO3a activity. Active FoxO3a then transcriptionally increases cav-1 level and cells undergo apoptosis via high Fas expression and caspase-3/7 activity. In contrast, PTEN activity is low in response to IPF fibroblast attachment to collagen matrix, which activates Akt thereby suppressing FoxO3a function. Cav-1 transcription is low due to inactive FoxO3a, thereby suppressing Fas expression & caspase-3/7 activity, which subsequently protects IPF fibroblasts from polymerized collagen-induced apoptosis.

In summary, we demonstrate that IPF fibroblasts display an apoptotic resistant phenotype on polymerized collagen. The mechanism for this resistance involves inactivation of FoxO3a by Akt which down-regulates cav-1 expression (Fig. 8). Decreases in cav-1 reduce Fas expression and promote resistance to apoptosis on polymerized collagen matrix. Based on our findings in this report, we suggest that the modulation of FoxO3 dependent cav-1/Fas specific functions may limit the progression of fibrosis and may be a useful approach for the treatment of this lethal disease.
